# Challenges and other linked features in promoting open access to bioinformation literature over about 2 decades

**DOI:** 10.6026/97320630018525

**Published:** 2022-06-30

**Authors:** Senkuttavan Pavithra, Pandjassarame Kangueane

**Affiliations:** 1 Biomedical Informatics (P) Limited, 17 Irulan Sandy Annex, Puducherry 607 402, India

**Keywords:** literature, open access, gold, bioinformation, decades, challenges

## Abstract

Open access to known literature is critical for creating a harmonious society across continents on planet earth. However, this objective is not simple. Therefore, it is of interest to document the challenges and linked features in promoting open access to bioinformation literature over about 2 decades.

## Open access (OA):

Access to available literature for advancement through the application of science for the society is secretly sensitive yet sacred. The quote from BOAI "the promise was that removing access barriers would allow the world to "accelerate research, enrich education, share the learning of the rich with the poor and the poor with the rich ... and lay the foundation for uniting humanity in a common intellectual conversation and quest for knowledge" explains everything as described elsewhere [1]. Thus, the formation of several open access (free to read) journals through the initiatives like PMC is engaging, entertaining and enlightening for everyone as listed elsewhere [2].

## Open access (OA) and Scientific community:

It is the responsibility of all stakeholders and beneficiaries especially the scientific community (advanced teaching and research) to make all reports available for the general public (tax payers (direct or indirect)). Please be informed that all the about 8 billion [3] people pay tax or render service for tax in one way or the other. Open access is a joint responsibility of all stakeholders in this context.

## Science, Scientist, Society and Service:

Science for service in a society is the success story of scientists with serenity as described elsewhere [4]. The commerce in science publishing is highlighted in this note. Science is not for the self success of scientists and it is for the society especially to serve the under privileged population. Please be informed that farmers and farm workers contribute to tax money (either direct or indirect) that is used for science and its advancement in all economies (developed, developing and under developed). Therefore, the research notes from science funded by tax payers should be made available and accessible to the kins of all tax payers. It should be noted that all members of the globe participate (direct and indirect) in a democracy through contribution with pride and respect.

## Author contributions in Biology:

Biology is a complex subject with many unknown facts. So there is a need to expand the scientific community across continents. Therefore, liberal engagement of scientific views across continents through open access publishing models is highly pertinent in the modern socio economic setup.

## Freedom of Press or Media:

We quote "It is the principle that communication and expression through various media, including printed and electronic media, especially published materials, should be considered a right to be exercised freely. Such freedom implies the absence of interference from an overreaching state; its preservation may be sought through constitutional or other legal protections" as described elsewhere [5].

## Role of Governments:

Role of government should be neutral by upholding freedom of the press (expression and human rights) and natural justice as per article 10 of the constitution of UK or article 19 of the constitution of India. Governments or representatives of governments should stay away from blocking Freedom of the Press (expression). Government notifications in any form in relation to as hindrance to the freedom of the press are unwarranted. It is often biased to certain parties and it is unconstitutional in nature. It should be noted that all publications are classified as New Papers under the GST schedule. Hence, biased notifications favoring certain parties in commerce are unwarranted. It should be noted that science for service in a society is the success story of scientists with serenity as described elsewhere [4]. Hence, engagement is essential.

## Academic Freedom:

It should be informed that academic freedom is an integral part of an academy where views and opinion of academicians should be entertained in a free and fair manner. This will accelerate discovery and innovations with improved service having optimal checks and balances where required with transparency. Members of the academy have tools such as the RTI and the Honorable ombudsman [6] in all known democracies with modern civilization within the ambit of the UN charter. Members should write to such authorities for such rights using a multi-faceted modus operandi to sustain natural justice. The role of heads (Directors, Vice Chancellors, and Presidents) of institutions (government or non-government) is critical even if voluminous (huge) in nature. Members of the academy should follow general guidelines (These are not hard and fast rules. This may be local in perspective) as per natural justice.

## Note on service:

It should be noted that everyone is working or providing service or rendering their skills for a price in a social democracy with pride, equality, freedom and liberty.

## Declaration by authors on publication ethics for submission:

The authors declare that they adhere with COPE guidelines on publishing ethics as described elsewhere [7]. The authors also undertake that they are not associated with any other second or third parties (governmental or non-governmental agencies) linking with any form of unethical issues or activities connecting to this publication. The authors also declare that they are not withholding any information that is misleading to the publisher in regard to their submission.

## Content Licensing:

This is an Open Access Journal which permits unrestricted use, distribution, and reproduction in any medium, provided the original work is properly credited. This is distributed under the terms of the Creative Commons Attribution License [8].

## Publishing Ethics:

The journal adheres to COPE guidelines on publishing ethics as described elsewhere [7]. It is the responsibility of the authors and especially the corresponding author to adhere to all forms of publishing ethics.

## Processing Speed:

Manuscripts are processed immediately within hours from the time of submission so as to make a final decision within 14 days after rigorous checking and editorial (and/or) peer reviews.

## Presentation of a manuscript:

### Writing Quality:

Writing should be highly critical, concise, coherent, precise, specific and direct to the context.

### Background data:

Each statement (except for the last 1 or 2 statements) made under background needs a valid and a closely relevant reference where possible. Please refrain (meaning avoid) from making general statements.

### Short conclusion:

Conclusion should be simple and direct in not more than 2-3 statements. Statements should be coherent (connect with one another).

### Plagiarism Check:

The journal uses several available search (TEXT Pattern Matching AND (OR) Natural Language Processing (NLP)) tools (COMMERCIAL and (or) FREE) enabled with representative databases. This is in addition to manual (editorial) screening through reading for adequate validation where possible. Please be informed that "inappropriate text recycling" in Abstract, Background, Results, Discussion, Conclusion, Table Captions and Figure Legends are NOT ALLOWED.

### Review Process:

All manuscripts submitted to BIOINFORMATION [9] are processed immediately using Scholar One (Clarivate Analytics, Inc) [10] and is subjected to editorial (ONE or more) and (or) "closely relevant peer" (ONE or more) reviews where required. Please be informed that Publons [11] has become an integral part of Web of Science and hence Scholar One uses Publons for reviewer selection in addition to author suggested reviewers.

### Timely Peer Review:

This is very important. Peer review is done on a voluntary basis thanks to the peer reviewers who entertain such services in the interest of data quality. Peer review should be fair, just and timely. Databases such as Publons in Scholar One (Clarivate Analytics, Inc) [10] help scrawl for hundreds of relevant peer reviewers automatically within minutes if not in seconds. Mixed reviews are a challenge to handling editors. Maximum reviews help the authors. However, maximum reviews often results in mixed reviews and views. This remains as a great challenge to publisher.

### Editorial role:

The role of editors is critical in journal publishing. This does not just remain in handling the manuscript by sending to two reviews and making a decision based on two reviews. This may not be justifiable in most cases. The role of editors is to help edit the manuscripts towards improvement and engage the scientific community. Editors usually have dual jobs. Their main role is academia where they draw their salary. Editor’s role is often voluntary and inclusive in nature. The degree of justice in such scenario is debated. Very few editors are 100% dedicated as editors in journal publishing. Reading is easy, writing is difficult and editing is noble.

### Comments and post publication reviews:

The journal welcomes comments, letter to the editors and post publication reviews as comments from readers.

### Open access (GOLD):

BIOINFORMATION is open access (GOLD).

### Open access (OA) contribution:

Open access (OA) contribution: An open access (OA) contribution (Production; indexing; CROSSREF DOI [12], open access charge; Editorial expenses and maintenance charges) is applicable. This helps readers around the world to download articles from BIOINFORMATION for free of cost. Please be informed that OA contribution is applicable only for those manuscripts found suitable for publication in the journal after administrative checking followed by editorial and (or) peer reviews. We provide adequate discount upon kind request where feasible and applicable.

### Waiver request:

Waiver request should be made during submission and it is possible to some extend through request and negotiation. Please be informed that 100% waiver is NOT possible as we are not funded by any Government agency.

### Donations:

The journal welcomes donations and contributions to support BIOINFORMATION, an open access publication. The readers can make voluntary contribution to the journal from as low as USD 1.

### Withdrawal policies:

ScholarOne online or other third party tools allows automatic withdrawal of manuscript before an initial and (or) a final decision is made for FREE. However, withdrawal is not automatically possible after an initial and (or) a final decision is made. These cases will be referred to the board (meets on a monthly basis) for a detailed discussion to take such decision where possible. It should be noted that a minimal processing fee is applicable for using the ScholarOne online software or other third party tools in such cases where the petition is not absolutely fair and (or) justified. Please be informed that this is not applicable for rejected manuscripts.

### Retraction policies:

The journal is fully open access. We take all possible steps to comply with publishing ethics as per COPE guidelines [7]. However, the features describing publishing ethics are often limitless in nature. Hence, we invite and entertain readers to report such issues to the publisher to analyze and investigate the corresponding data to take appropriate decision after a detailed discussion in a transparent manner.

### Advisory Note:

Authors are advised to check their manuscript for any unacceptable degree of TEXT recycling using tools as described elsewhere [13- 15] and made available at the host institute. Please also upload a copy of such reports during submission to avoid delay in processing.

### Discount offer:

Discount offer is available for authors who provide self plagiarism check report using tools as described elsewhere [13- 15] during submission. Please check with your institution for the availability of such tools. This will help speed up processing.

## SCOPUS:

The SCOPUS database [16] is a product of a major science and social journal publisher company named Elsevier Inc [17] from Netherlands. The linked company is RELX INDIA PRIVATE LIMITED (CIN: U72900DL1996PTC077903) with Authorised Capital (INR) 177,10,00,000 and Paid up Capital (INR) 154,63,28,520 as per MCA portal [18] and wikipedia [19] as on March 31, 2020. Elsevier plays dual role both as a publisher and indexing resource creator. This position of Elsevier Inc from Netherlands is clearly biased and ambiguous in nature. There is commerce involved and academia is misinformed by this position followed by their unwarranted marketing strategies within academia. Freedom of press is primary. There are COPE guidelines to balance data of all form in open access publishing models. The attempted service provided by Elsevier Inc is exhaustive and therefore, the board taking such decision should be transparent, comprehensive, representative and exhaustive to uphold the freedom of the press and natural justice. A couple of dozen people should not make such decision to influence commerce at large across continents covering over about 8 billion people. Names and credibility of the board should be transparent and listed in both online and offline media. It is in the interest of transparency that the "turn-around time" to address and entertain any issue raised by the "so called" board (usually latent) should be less than 14 days and clearly not 3 or 4 years. The message with such intention is clearly biased and commercial in nature. Please be advised that commerce is a function of time. Justice delayed is justice denied. It should be noted that we credit Elsevier Inc. for maintaining Science Direct [20] for a price though commercial. The efforts to make contents in Science Direct accessible worldwide will be clearly noble. Please be informed that commerce is a part of economics around the world. It is also not fair to dominate the space through monopoly aided by capitalism sponsored by banks in support of states. Grow and let others to grow too in a world dominant with democratic socialistic principles. Teach them, help them, embrace them and enlighten them where possible if you are in a better position. This should be all inclusive.

## EBSCO:

EBSCO [21] is an indexing database provider from the USA. It is neutral in its operation that is fair and just. However, it sponsors the DOAJ [22] database that is latently linked to a Sweden based Lund University [23] with some unknown links to organizations in Netherlands.

## Springer Nature Inc.

Springer Nature [24] (with several firms registered in India under different names as per MCA portal [18]) is a major science publisher from Germany and or Switzerland. The position taken by Springer Nature as a publisher in the open domain is unbiased. However, it is linked with a number of societies in Europe through "potential sponsor" related associations.

## Web of Science:

Web of Science is a product of about 3.55 billion USD with about 100 million USD 2019 revenue (November 14, 2020; WIKI data [19]) Clarivate Analytics, Inc. [10] (Clarivate Analytics (India) Private Limited with CIN: U74999MH2016FTC283853 under Mumbai ROC, MCA India [18] having with an authorized Capital of INR 50,00,000 as on November 14, 2020) a data analytics company based in the USA. The position taken by Clarivate Analytics, Inc is unbiased. However, Clarivate Analytics, Inc as private entity influence commerce from academia through capitalism and listing of all known journals using transparent metrics (optimal metrics which is neither conservative nor liberal) is highly warranted. In other words all journals after 5 years (or 3 years as in previous precedence) since its launch should be given an Impact Factor [25] where possible in the interest of natural justice. Let the community make the decision and private parties should restrict themselves within certain limits where possible in the interest of transparency. Ranking with transparent metrics is acceptable. However, cut-off is not acceptable and it is unfair. This is not engagement. The service provided by Clarivate Analytics, Inc is exhaustive and therefore, the board taking such decision should be transparent, comprehensive, representative and exhaustive to uphold the freedom of the press and natural justice. A couple of dozen people should not make such decision to influence commerce at large across continents covering over about 8 billion people. Names and credibility of the board should be transparent and listed in both online and offline media. It is in the interest of transparency that the "turn-around time" to address and entertain any issue raised by the "so called" board (usually latent) should be less than 14 days and clearly not 3 or 4 years. The message with such intention is clearly biased. Please be advised that commerce is a function of time. Justice delayed is justice denied. A company capable of analyzing such huge data in a many to many map model should be all inclusive and most transparent towards upholding the Freedom of the Press which is fundamental to any other debate in this regard. It should be noted that data from the under developed parts of the world is highly relevant for the advancement of science. This is not possible without a broad spectrum of engagement.

## Note on predatory (meaning greedy) publications (meaning journals):

It is a pointless pondering on predatory (meaning greedy) publications (meaning journals) while practicing publishing through freedom of the Press. It should be noted that a weak publication will vanish itself in an open access publishing model where contents are made available for free on the WWW. The fundamental question in this context is the definition of host and predator. The second question is the type (data and or commercial) and subsequent measure of effect of the predator on the host. Detailed discussion on this issue or any other related issue is welcomed under the freedom of the Press yet conclusion on it will be often biased and is clearly unwarranted. The parties aware of such concerns should write to the publisher (with address for communication) to take such action within such time to stand corrected. Please be informed that ISSN [26] is unique for each publication and portals for ISSN is distributed throughout the world in each country. This is well monitored and clearly streamlined. Therefore, NO two publication titles will be identical. Awareness from authors on misleading or misinformed or misrepresented ISSN is important and such information should be petitioned to ISSN and portals for ISSN that are distributed throughout the world with state mechanisms to monitor such activities. Academia should be self aware on these issues and have discussions on the quality and quantity of data taken to the context. Caveat Emptor is applicable among the literate community as in this case to a considerable extend. The only problem could arise because of compromised (unregistered or mirrored) ISSN number published on the WWW which is already well regulated through DNS lookup. Therefore, parties concerned about ethical issues on scientific publishing should write to concerned publishers with known address to stand corrected or to ISSN and portals for ISSN or to DNS [27] lookup where address is not available to correct such issues through available state mechanisms. Hence, biased advisory notes from government representations, society sponsored mass campaign through news/TV media and academic miss representation based on data collected by an individual without physical address for communication is clearly unwarranted in this regard.

## Conclusion:

We thus document the challenges and linked features in promoting open access to bioinformation literature over a period of about 2 decades.

## About the authors:

Pandjassarame Kangueane [28] is a scientist, an author of scholarly materials, teacher of higher education in Biotechnology and Bioinformatics, Professor, educationalist, editor, journalist, entrepreneur, social reformer, and a farmer. He hopes to bring smile in the face of the under-privileged in every possible way. He received his Bachelor of Technology in Industrial Biotechnology from Anna University (1997), India and Doctor of Philosophy in Bioinformatics from the National University of Singapore (2001), Singapore. He served as scientist, S*Bio Pte Ltd, Singapore (2001), visiting scientist, Chiron Corporation Inc, Emeryville, California, USA (2001), Assistant Professor, Nanyang Technological University, Singapore (2002-2006), Director, Biomedical Informatics Private Ltd, India (2001-till date), Visiting Professor, VIT University, India (2007-2009), Professor, AIMST University, Malaysia (2009-2011), Chief Editor, Bioinformation, India (2005-till date) and Associate Editor, BMC Bioinformatics, Biomed Central, United Kingdom (2007-till date). He is an active farmer cultivating paddy, sugarcane, black grams, green grams, coconuts and vegetables.

Senkuttuvan Pavithra received her Bachelor of Commerce (2016) and Master of Commerce (2018) from Pondicherry University, Union Territory of Pondicherry (a French colony of pre independence India), India. She is an alumni of the alma mater Saradha Gangadharan College, Pondicherry 600 5004 which is affiliated with Pondicherry University. She serves as production director at Biomedical Informatics (P) Ltd since 2018.
